# Bioinformatics investigation on blood-based gene expressions of Alzheimer's disease revealed ORAI2 gene biomarker susceptibility: An explainable artificial intelligence-based approach

**DOI:** 10.1007/s11011-023-01171-0

**Published:** 2023-02-21

**Authors:** Karthik Sekaran, Alsamman M. Alsamman, C. George Priya Doss, Hatem Zayed

**Affiliations:** 1 Laboratory of Integrative Genomics, Department of Integrative Biology, School of BioSciences and Technology, Vellore Institute of Technology (VIT), Vellore, 632014 Tamil Nadu, India; 2 Department of Genome Mapping, Molecular Genetics and Genome Mapping Laboratory, Agricultural Genetic Engineering Research Institute, Giza, Egypt; 3 Department of Biomedical Sciences College of Health Sciences, QU Health, Qatar University, Doha, Qatar

**Keywords:** Alzheimer's, Artificial Intelligence, Biomarkers, Genetic Algorithm, Machine Learning, *ORAI2*, *STIM2*

## Abstract

The progressive, chronic nature of Alzheimer's disease (AD), a form of dementia, defaces the adulthood of elderly individuals. The pathogenesis of the condition is primarily unascertained, turning the treatment efficacy more arduous. Therefore, understanding the genetic etiology of AD is essential to identifying targeted therapeutics. This study aimed to use machine-learning techniques of expressed genes in patients with AD to identify potential biomarkers that can be used for future therapy. The dataset is accessed from the Gene Expression Omnibus (GEO) database (Accession Number: GSE36980). The subgroups (AD blood samples from frontal, hippocampal, and temporal regions) are individually investigated against non-AD models. Prioritized gene cluster analyses are conducted with the STRING database. The candidate gene biomarkers were trained with various supervised machine-learning (ML) classification algorithms. The interpretation of the model prediction is perpetrated with explainable artificial intelligence (AI) techniques. This experiment revealed 34, 60, and 28 genes as target biomarkers of AD mapped from the frontal, hippocampal, and temporal regions. It is identified *ORAI2* as a shared biomarker in all three areas strongly associated with AD's progression. The pathway analysis showed that *STIM1* and *TRPC3* are strongly associated with *ORAI2*. We found three hub genes, *TPI1, STIM1,* and *TRPC3*, in the network of the *ORAI2* gene that might be involved in the molecular pathogenesis of AD. Naive Bayes classified the samples of different groups by fivefold cross-validation with 100% accuracy. AI and ML are promising tools in identifying disease-associated genes that will advance the field of targeted therapeutics against genetic diseases.

**Supplementary Information:**

The online version contains supplementary material available at 10.1007/s11011-023-01171-0.

## Introduction

Alzheimer's disease (AD) is a neurodegenerative disorder that causes brain atrophy and eventually destroys brain cells (Al-Thani et al. 2021). The progression of AD involves cognitive impairments such as memory loss, delusion, disorientation, and confusion. The shrinkage of blood vessels and muscles, inflammation, mitochondrial dysfunction, and production of free radicals are a few reasons that trigger AD (Chethana et al. 2022). AD seems to have a vital genetic component, emphasizing the potential of developing targeted novel therapies to treat AD. According to the National Institute on Aging (NIA), AD is directly linked to Apolipoprotein E (*APOE*) gene, which triggers AD by disrupting the blood–brain barrier (BBB) integrity (Koutsodendris et al. 2022). However, this gene is not the primary cause of all AD cases. The unknown gene–gene/environment interactions complicate understanding the direct cause of AD. According to age, Alzheimer's is primarily divided into two categories, early-onset and late-onset, where no specific gene is found to impact the disease progression. Recent studies identified a significant association between mutated *APP*, *CD33*, and *BIN1* genes and the development of AD and neurodegenerative comorbidities (Bhattacharyya et al. 2022).

According to the special report from Alzheimer's Association, 2022, cognitive assessments with the availability of potential blood-based gene biomarkers to aid in early detection and better AD diagnosis. Early AD diagnosis significantly reduces the effect and improves treatment outcomes quickly**.** This fact exemplifies the potential of gene-based treatment methods that often increases treatment efficacy compared to traditional methods. Several studies have identified several potential biomarkers for AD in recent years. The whole genome sequencing technologies were used to investigate the relationship between cognition-related traits and 174 polymorphisms located on *CD36*. Six genetically linked variants in the *CD36* gene were found to significantly delay the onset of AD (Sery et al. 2022). A ligand library containing 60 natural compounds retrieved from the literature and 25 synthetic compounds from DrugBank is screened for validation against the AD markers. Molecular docking tests identified 11-oxo-tigogenin as the most significant ligand molecule with a binding affinity of -11.1 kcal/mole forming three hydrogen bonds with Arg176, Trp124, and Ile174 (Kushwaha et al. 2021).

The extensive growth in bioinformatics opened up many possibilities for new dimensions of clinical applications (Doss and Zayed 2017; Zaki et al. 2017; Ebrahimi et al. 2018; Thirumal Kumar et al. 2018); Sekaran K et al. 2021). In digital format, multitudinous medical data provide precise insights into any disease. Machine learning (ML) propounds advanced algorithms to solve complex problems in critical domains. In healthcare systems, ML models are widely used to perform clinical diagnosis, biomarker identification, tumor identification, and drug target discovery for various diseases. For instance, since the outbreak of the COVID-19 pandemic, many *in-silico* studies have been conducted to find novel therapeutics as it takes less time and physical compounds than wet-lab experiments (Bagabir et al. 2022; Yang et al. 2022). The machine learning method Artificial Neural Network (ANN) has been tested to identify the biomarker pattern of Alzheimer's disease. This method predicted gene interaction using a continuous stepwise algorithm and identified fifty potentially influential AD genes in the hippocampus region (Zafeiris et al. 2018). In order to detect capable genes from microarray data and classify AD tissues into different classes, researchers propose a wrapper-based feature selection technique that combines the genetic algorithm with support vector machines (SVM) (Scheubert et al. 2012). This method identifies the twenty most promising candidate markers with three common genes.

Although machine learning (ML) techniques help identify potential disease-associated biomarkers, they require highly developed computational abilities to evaluate their potentiality. Additionally, increased performance is frequently achieved through increased model complexity, transforming such systems into "black box" methods and resulting in uncertainty about how they function and, ultimately, how they make decisions (Linardatos et al. 2020). It is challenging to rely on models whose conclusions cannot be clearly understood. Specific to the medical domain, the AI based clinical decisions about treatment options for a particular condition strongly impact individuals’ well-being. The field of explainable Artificial Intelligence (XAI), which focuses on understanding and interpreting AI systems' behavior, has resurfaced due to the demand for reliable, equitable, robust, high-performing models for real-world applications (Gunning 2019). XAI was used to identify squamous cell carcinoma biomarkers using ML models trained on binary classification datasets containing expression data from healthy and cancer skin samples. Following successful incorporation, 23 significant genes associated with skin cancer progression were discovered, which might serve as diagnostic and prognostic biomarkers. XAI techniques demonstrated that the model output was interpretable by establishing a relationship between the model output and the relevant genes. The explainable clustering and classification approach was used to find and interpret age-based differences in brain tumor diseases (Meena and Hasija 2022). The increased use of ML-based XAI approaches in medical diagnosis has sparked the interest of many future researchers seeking to implement these effective methods in complex diseases.

There is a need to use these advanced computational methods to provide healthcare with valuable biomarkers for early AD detection. Such markers can also shed light on the complex gene network of Alzheimer's pathogenicity. This paper proposes a bioinformatics investigation to identify AD's gene biomarkers from the blood-based gene expression data. We attempt to use gene expression analysis to reduce the dimension of the data statistically and simplify the candidate selection process. The genetic algorithm scrutinizes the DEGs to remove irrelevant features from the subset. The performance and significance of machine learning algorithms trained with extracted biomarker genes will be assessed. The trained models will be interpreted using XAI techniques. Pathway modeling, functional and pathway enrichment, and protein interactions will be performed on the strongly associated genes of identified AD biomarkers.

## Materials and methods

### Data analysis pipeline

**Fig. 1:**
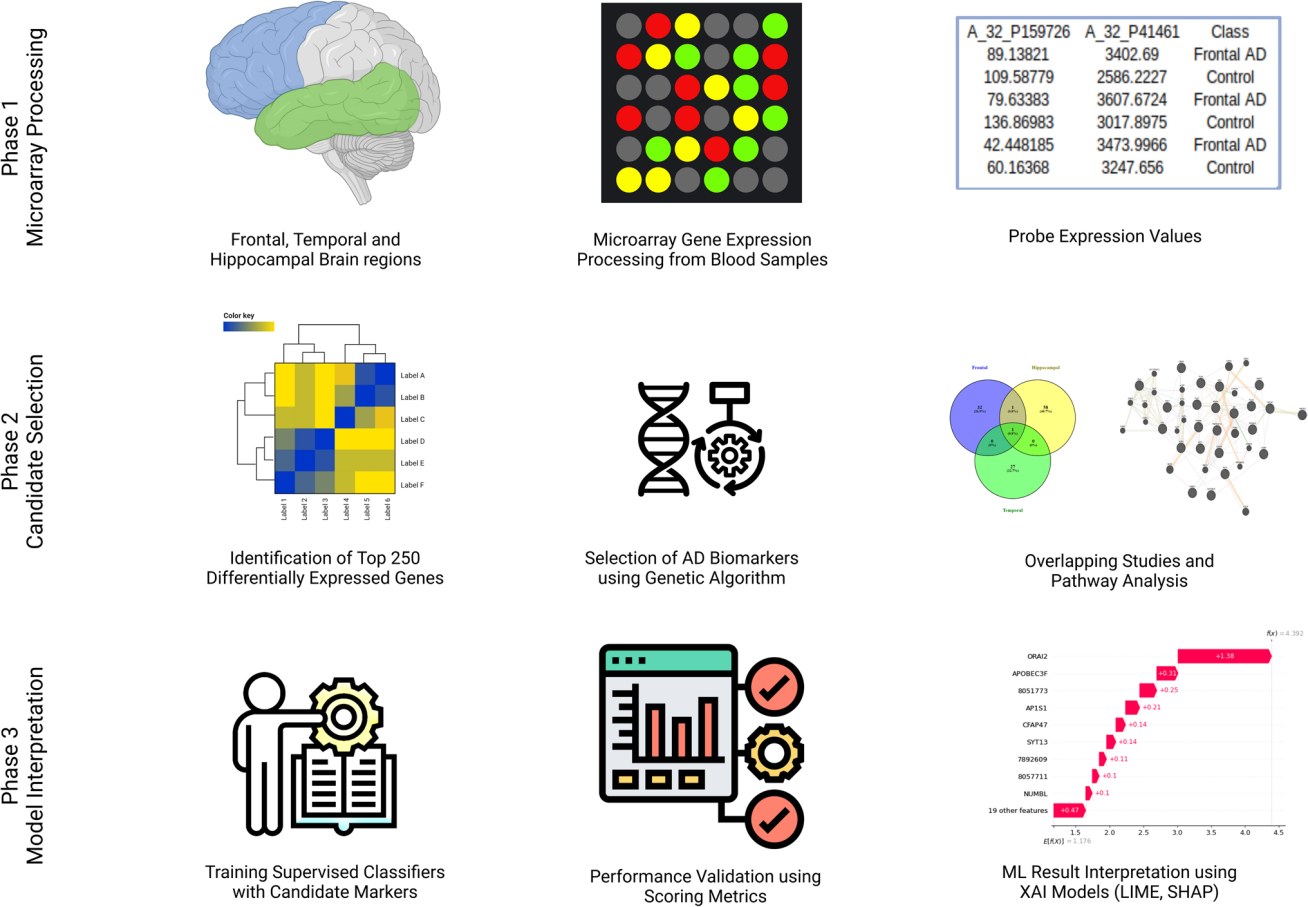
Data analysis pipeline used in this study. The gene expression data analysis (phase I) and AD biomarker selection using genetic algorithms (phase II). Machine learning, model training, and results interpretation (phase)

This study aimed to investigate DEGs in patients with AD and identify candidate biomarkers through statistical and machine-learning techniques that might be relevant for treating AD. RNA samples from the blood tissue were collected from three brain regions frontal, temporal and hippocampal. The experimental design of the proposed study includes the following subsections—DEG selection criteria discuss the statistical gene selection procedure, Genetic Algorithm identifies candidate biomarkers of AD through the synthesized natural evolutionary process. The Cytoscape tool performs the gene co-expression analysis. ML algorithms are trained and validated with the gene subset under many steps involved in the task. Explainable artificial intelligence techniques delineate the interpretation of the trained models. Figure 1 visualizes the order of phases involved in the proposed system. This pipeline is implemented in Python (Anaconda Distribution) with supportive ML libraries and XAI modules. The source code of the article is available at https://github.com/karthiksekaran/alzheimer-biomarker-study-XAI.

### Gene expression data collection and analysis

The NCBI-GEO database (http://www.ncbi.nlm.nih.gov/geo/) was used to obtain gene expression data. Hokama et al. (2014) experimental data with accession number GSE36980 was used in this study to train and evaluate the ML models. This data set contains array-based expression profiling data for three different brain regions: frontal (33 samples), hippocampal (18 samples), and temporal (29 samples). This experimental study looked for genes that differed between AD and healthy individuals (Case and Control based study). The credible way to select useful biomarkers in gene expression profiling is by conducting differential gene expression analysis. GEO2R was used to compare two groups of gene expression data, healthy and AD cases, across three different brain regions samples. GEO2R is an interactive web platform supported by NCBI to compare two or more groups of samples available in the GEO series to find DEG from the expression data (Barrett et al. 2012). The DEGs are often expressed in an irregular pattern between two experimental conditions when they are statistically significant. The adjustment is made to the p-value through the Benjamini and Hochberg false discovery rate method. Log transformation is applied, and the typical values are replaced with logarithmic values. This technique normalizes the dataset by addressing skewness trouble. We identified the top 250 DEGs with GEO2R, irrespective of the p-value scale, to avoid rejecting the informative AD biomarkers. Furthermore, to reduce the data used for machine learning model training and to increase diversity.

### Biomarker selection using a genetic algorithm

The intrinsic impediment in gene-based computer modeling is finding the most prominent biomarkers from inordinate dimensions of genome information. The genetic algorithm analysis method is widely used to solve search-related optimization problems by performing biological operations such as selection, crossover, and mutation (John 1992). In this study, the genetic algorithm method was used to reduce the high dimensionality of the gene expression data to obtain the optimal subset of features for ML model training. Weka 3.8.2, a java based machine learning software, is used to perform feature selection from the attribute search options. The GA algorithm identified 34, 60, and 28 from frontal, hippocampal, and temporal regions. Based on the observation, the *ORAI2* gene was found in all three regions, whereas the *TPI1* gene is present only in common between frontal and hippocampal regions. Further experiments on the identified novel AD markers disclosed many associated genes (Table 1). The hypothesis of GA was demonstrated with few requirements, a genetic representation of the solution and a fitness function to evaluate the solution (Sayed et al. 2019).

**Table 1:** Top 10 ranked genes associated with ORAI2 generated through genemania

Gene	Description	Rank
ORAI3	ORAI calcium release-activated calcium modulator 3	1
ORAI1	ORAI calcium release-activated calcium modulator 1	2
CRACR2A	calcium release activated channel regulator 2A	3
TRPC3	transient receptor potential cation channel subfamily C member 3	4
STIM1	stromal interaction molecule 1	5
RELT	RELT TNF receptor	6
SLC1A4	solute carrier family 1 member 4	7
FAAP100	FA core complex associated protein 100	8
PTGES2	prostaglandin E synthase 2	9
FBXO46	F-box protein 46	10

Scores of machine learning classifiers on GA biomarkers Metrics

### Pathway analysis and protein clustering

**Fig. 2:**
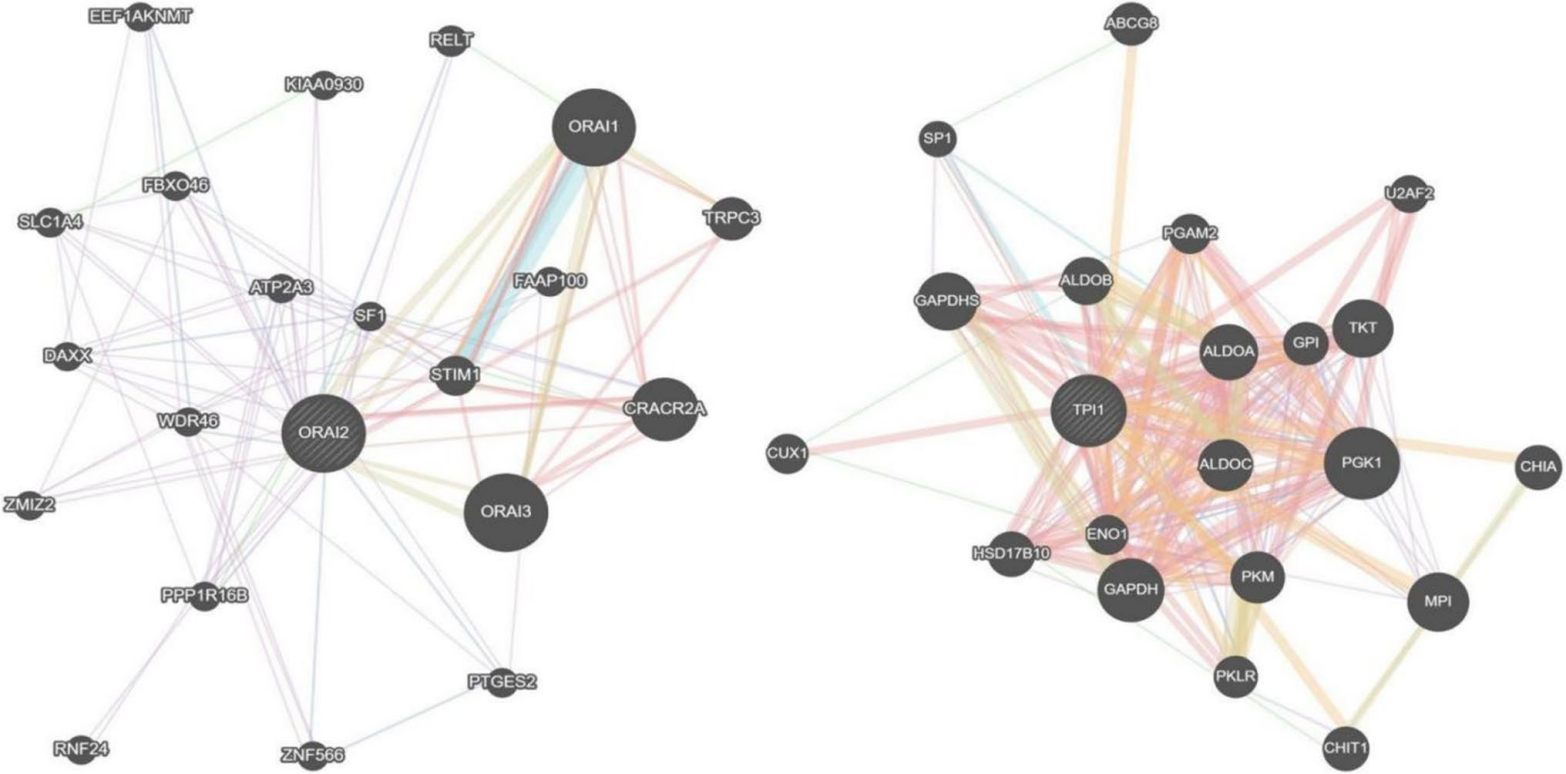
The protein–protein interaction between *ORAI2, TPI1* and the coalition genes was identified through the genetic algorithm method

The protein–protein interaction between the primary *ORAI2 gene*, secondary *TPI1*, and the coalition genes (Table 1) is mapped using the STRING database (Mering et al. 2003). Gene nodes further group the network analysis with k-means clustering of 3 distinct cluster units. In cluster 1, *ITPR3* and *TRPC6* are grouped, *ORAI2*, *STIM2* in cluster 2, and *ITPR1* (Lim et al. 2021), *ITPR2*, *ORAI1*, *ORAI3*, *STIM1, TRCP1*, and *TRPC3* forms cluster 3. All the network genes belong to *ITPR, ORAI, STIM,* or *TRP*. Similarly, the *TPI1* network is generated with the same number of clusters. *ALDOA, ENO1, GAPDH*, *GAPDHS, GPI, PGK1, PGK2,* and *TP1* form cluster 1, *TALDO1* and *TKT* belong to cluster 2, and *PGM1* alone make cluster 3. The GeneMANIA Cytoscape plug-in (Montojo et al. 2010) predicts the gene function for the root markers *ORAI2* and *TPI1*. The most related gene groups are identified by finding the association with their targets. Figure 2 depicts the interactions between *ORAI2* and *TPI1* with correlated markers.

### Machine learning and modeling

Supervised machine learning classification algorithms are trained with the biomarkers to find the discriminative pattern for classifying the samples of different subgroups. Besides, explainable artificial intelligence techniques such as Local Interpretation and Model Explanations (LIME) and SHapley Additive exPlainer (SHAP) are employed to interpret the model predictions (Lundberg and Lee 2017; Covert et al. 2021). The 250 DEGs identified by statistical analysis and 34 frontal, 60 hippocampal, and 28 temporal subsets identified using the genetic algorithm are used to train ML classifier models. The class label is represented in binary format (0-Case and 1-Control). Logistic Regression (LR), Random Forest (RF), Linear Support Vector Machines (L-SVM), Naive Bayes (NB), and Multilayered Perceptron Neural Network (MLP-NN) algorithms are used in this experiment. The sample size is not evenly distributed in each dataset, so a fivefold cross-validation technique is applied for model validation. The dataset is minimally imbalanced with the target classes. The performance of the trained models is evaluated with accuracy, precision, recall, F1-score, Matthew's correlation coefficient, and receiver's operational characteristic (ROC) curve to avoid evaluation bias.

### Explainable artificial intelligence

The essence of XAI is articulated by enunciating the methods and process of machine learning models to human dilettante (Antoniadi et al. 2021). XAI techniques are employed to describe the model predictions, the outcomes, and the biases. The characterization of model transparency, accuracy, and fairness becomes viable with XAI. In this study, the evaluation of the discriminative capacity of ML models is implemented by Local Interpretable Model-Agnostic Explanations (LIME) and SHapley Additive exPlanation (SHAP). These two XAI methods are model-agnostic and post hoc, applied after the model has been trained. LIME interprets ML models' predictions and remains model-independent by perturbing the input data. Observing the resulting impact on the output and the interaction with the local fidelity provides more information on individual predictions. While in the SHAP model, Shapley values explain the individual predictions (Vollert et al. 2021). The technique explains the model output by computing the individual feature contribution. The Shapley values are computed by coalitional game theory (Sekaran and Shanmugam 2022).

## Results

**Fig. 3:**
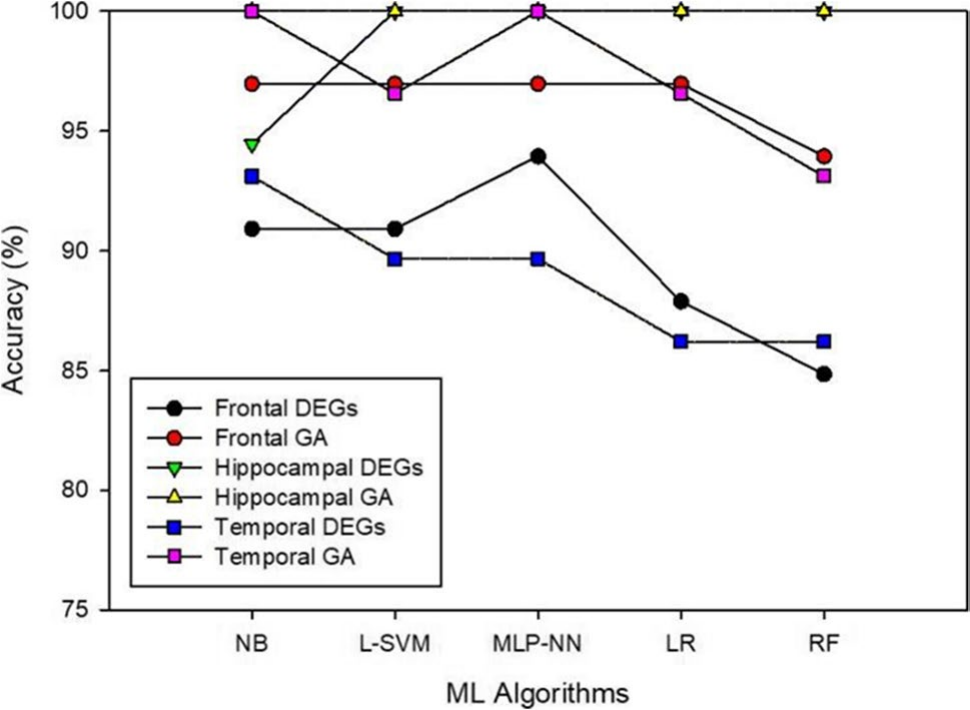
Accuracy of machine learning models on the DEG and GA features of 3 datasets

**Fig. 4:**
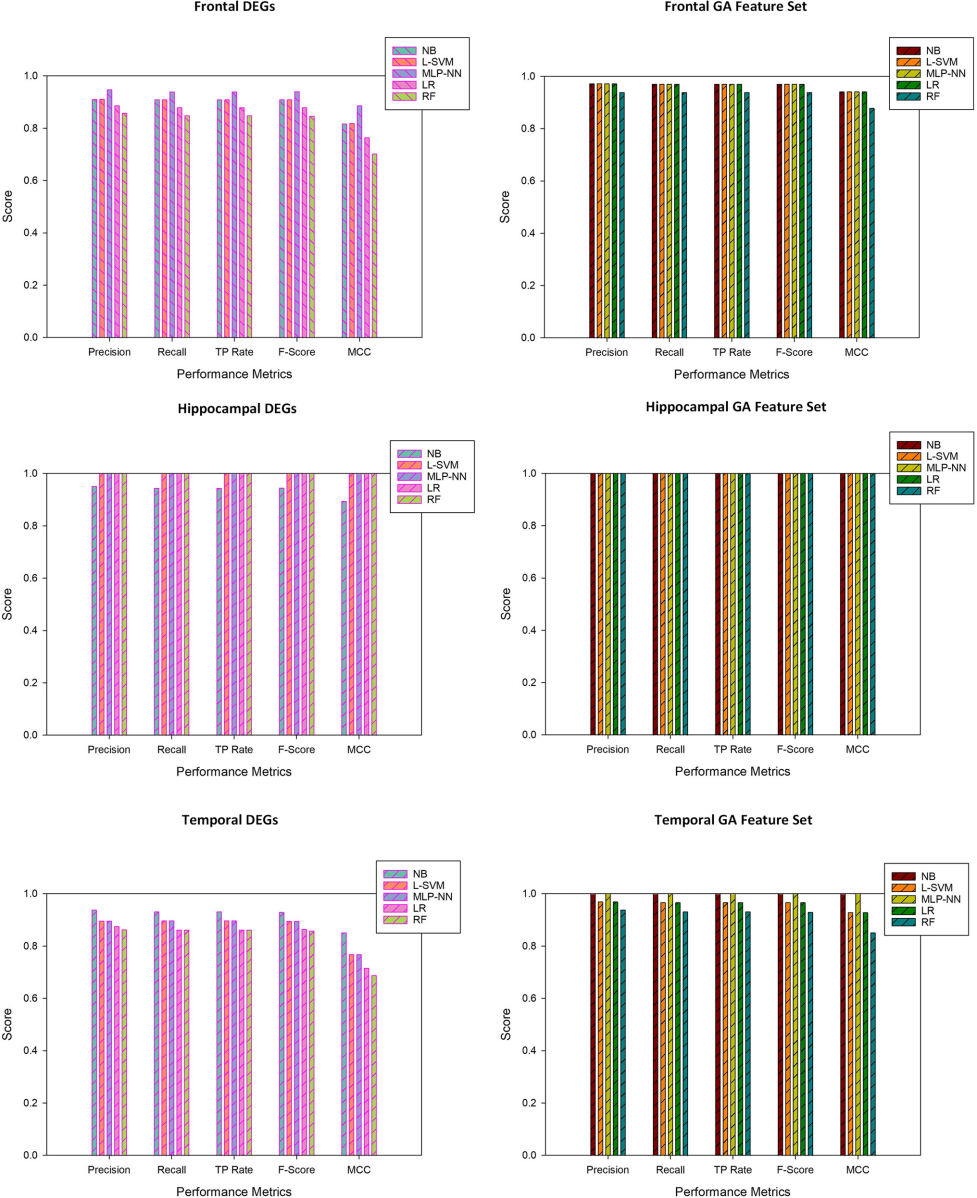
Performance of machine learning models on sample groups of Alzheimer's dataset

The scores of individual ML classifiers attained on candidate markers identified by the genetic algorithm are given (Table 2 and Fig. 3). Table 2 contains the outputs of frontal, hippocampal, and temporal datasets. The results have shown that the performance of NB, L-SVM, MLP-NN, and LR are the same on the frontal, but all five classifiers attained 100% performance on the hippocampal dataset. NB and MLP-NN outperformed other classifiers on the temporal dataset by scoring 100% output. Figure 4 illustrates the accuracy of ML models on DEG and GA subsets. In Fig. 3, the Naive Bayes algorithm stands out from the other classifiers with top performance. Figure 4 represents the performance of ML classifiers on three datasets. The graphs depict the scores of each classifier, and almost all attained better results.

**Table 2:** Scores of machine learning classifiers on GA biomarkers

Metrics	NB	L-SVM	MLP-NN	LR	RF	Dataset
Negative Predictive Value	94.44%	94.44%	94.44%	94.44%	94.44%	frontal
	100.00%	100.00%	100.00%	100.00%	100.00%	hippocampal
	100.00%	94.74%	100.00%	94.74%	100.00%	temporal
Positive Predictive Value	100.00%	100.00%	100.00%	100.00%	93.33%	frontal
	100.00%	100.00%	100.00%	100.00%	100.00%	hippocampal
	100.00%	100.00%	100.00%	100.00%	80.00%	temporal
Sensitivity	93.75%	93.75%	93.75%	93.75%	93.33%	frontal
	100.00%	100.00%	100.00%	100.00%	100.00%	hippocampal
	100.00%	90.91%	100.00%	90.91%	100.00%	temporal
	100.00%	100.00%	100.00%	100.00%	94.44%	frontal
	100.00%	100.00%	100.00%	100.00%	100.00%	hippocampal
	100.00%	100.00%	100.00%	100.00%	90.48%	temporal
Accuracy	96.97%	96.97%	96.97%	96.97%	93.94%	frontal
	100.00%	100.00%	100.00%	100.00%	100.00%	hippocampal
	100.00%	96.55%	100.00%	96.55%	93.10%	temporal

**Fig. 5:**
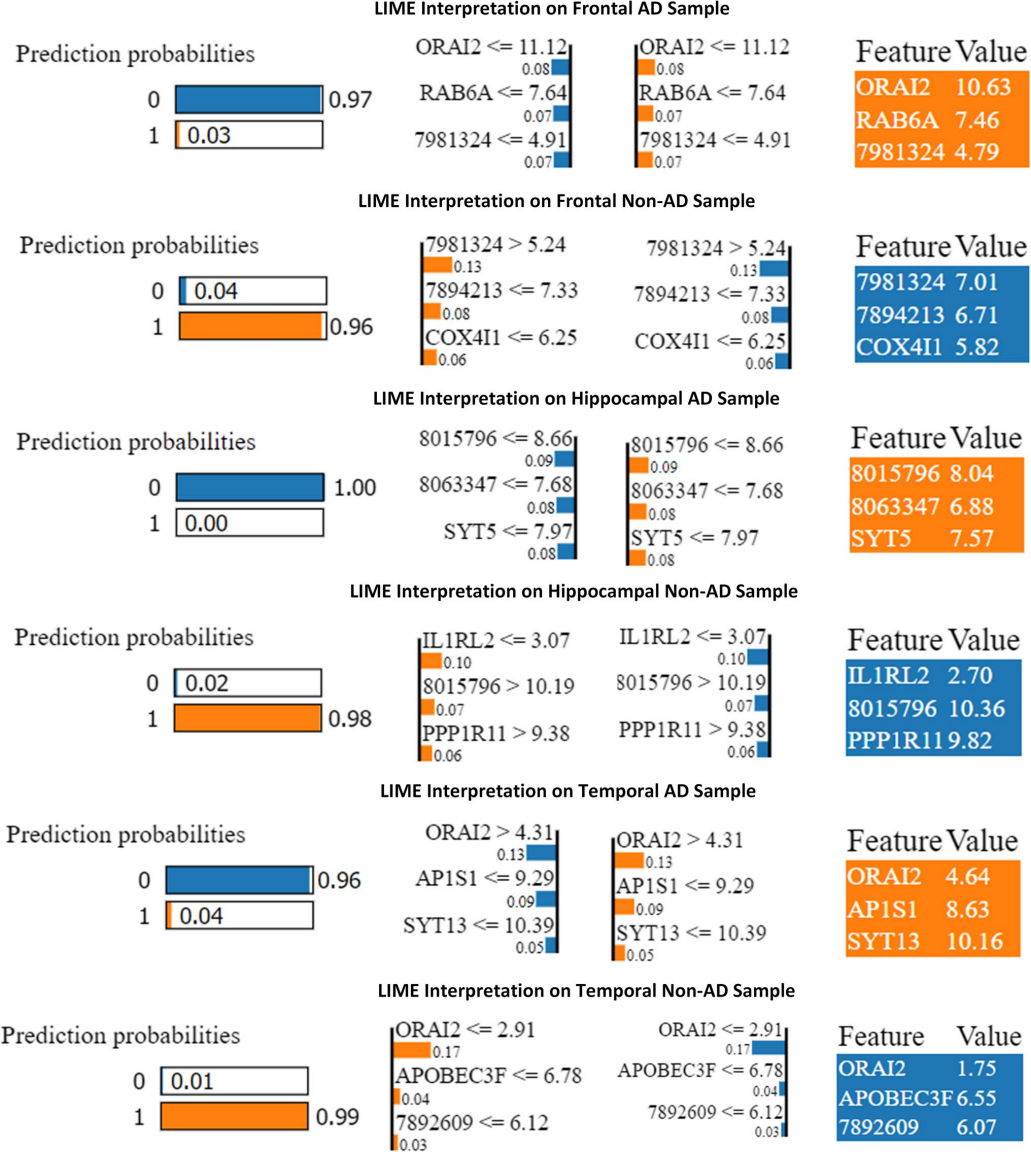
LIME interpretation on samples of Alzheimer's disease

The machine learning classifier's predictions were analyzed in the next phase with LIME and SHAP XAI models. Logistic regression is trained with the GA-identified subsets with 75% training data. In Fig. 5, two samples, each from frontal AD and non-AD, are randomly selected to test the predictions of the trained ML model. The prediction probability indicates the chances of being predicted as either class based on the features. *ORAI2*, *RAB6A*, and 7,981,324 are the top three biomarkers identified by LIME to predict frontal AD samples, and 79,814,324, 7,894,213, and *COX4I1* for frontal non-AD samples with 0.97 and 0.96 probability respectively. The feature value represents the importance of the particular feature in the predictions. The ranges given in the graph provide the conditions upon which the classifier makes the decision.

**Fig. 6:**
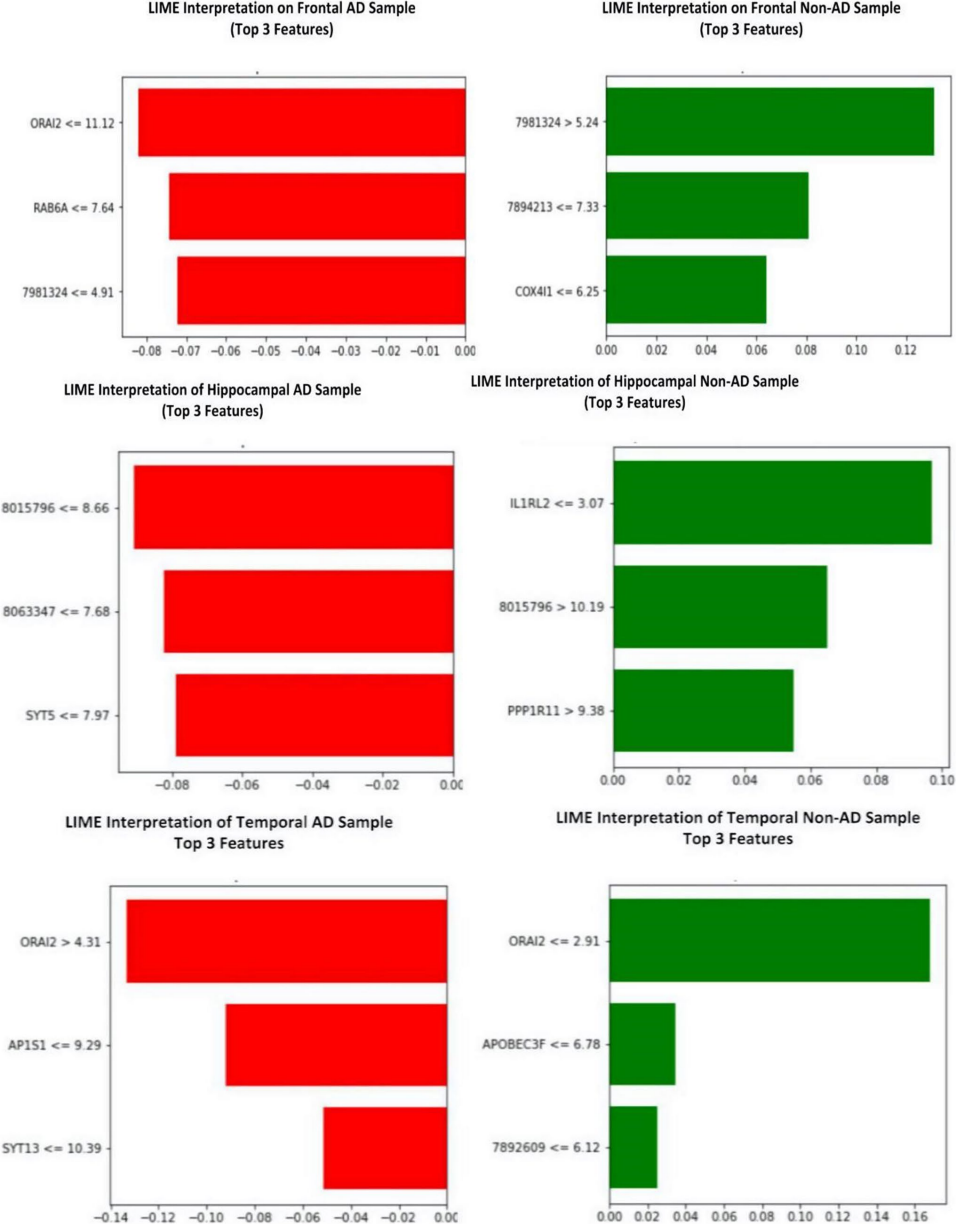
Top 3 gene scores of LIME interpretation on group samples of Alzheimer's disease

**Fig. 7:**
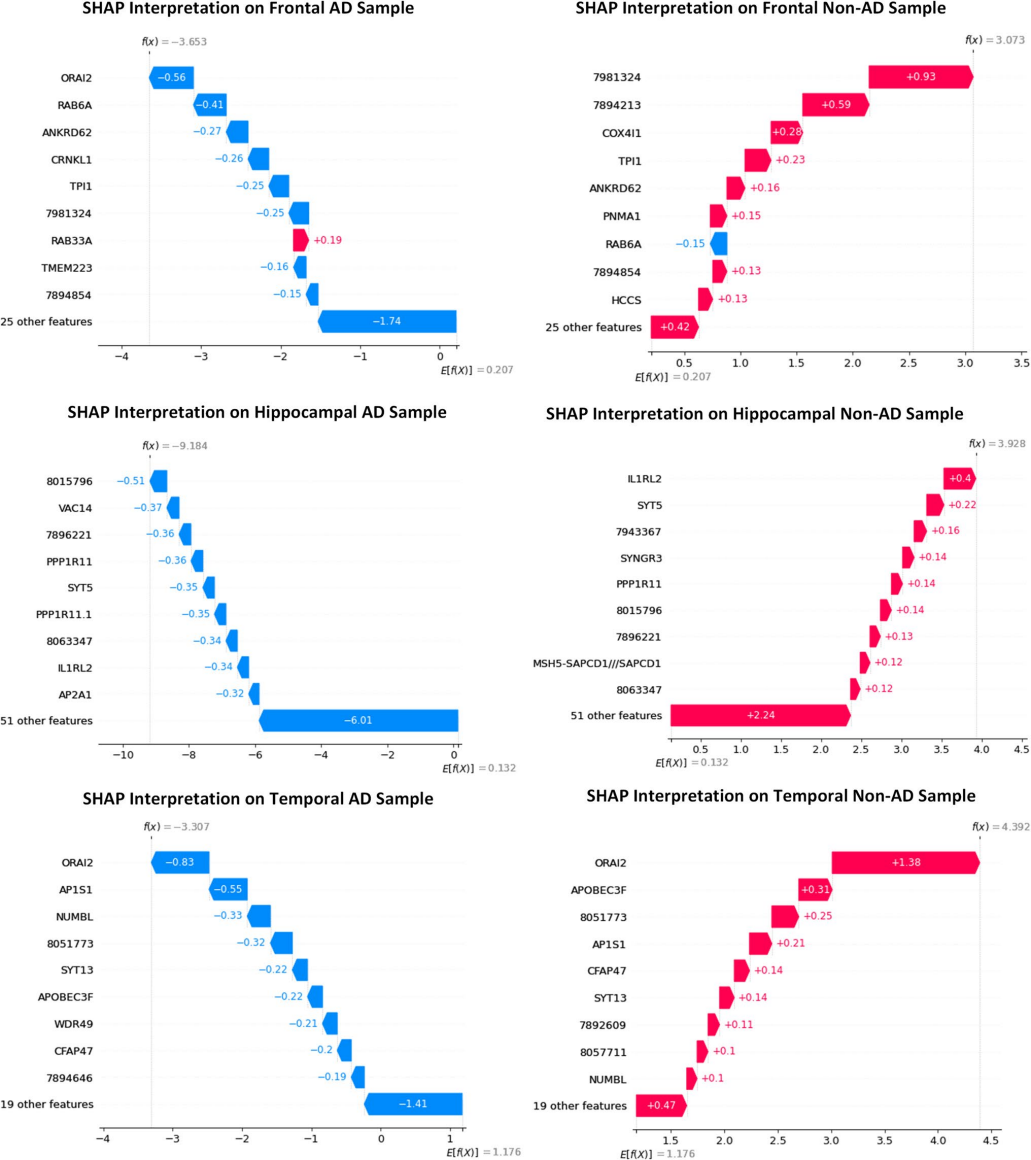
SHAP interpretation on samples groups of Alzheimer's disease

Similarly, 8,015,796, 8,063,347, and *SYT5* were the robust biomarkers for the hippocampal AD sample prediction with one probability, and *IL1RL2*, 8,015,796, and *PPP1R11* for hippocampal non-AD sample prediction with a probability score of 0.98. *ORAI2*, the crucial biomarker identified in this study, is an essential factor in predicting the temporal AD and non-AD samples alongside *AP1S1, SYT13, and APOBEC3F*, 7,892,609, respectively. The prediction probabilities of both classes from these genes were 0.96 and 0.99. Figure 6 represents the top three genes from each dataset. SHAP scores on the datasets reinforce the result attained by LIME. In Fig. 7, the negative gene values positively impact the AD prediction, while positive gene values on non-AD prediction. The negative SHAP value in blue represents AD (labeled as 0), while the positive in red denotes non-AD (labeled as 1) classes on all three datasets. The *ORAI2* gene is a decisive factor in classifying AD samples as 0 and the gene 7981324 as 1 on frontal AD and non-AD samples, respectively. Figure 7 shows the SHAP interpretation on hippocampal AD and non-AD samples, identifying 8,015,796 and *IL1RL2* (Luís et al. 2022) as the top contributors to the predictions. The *ORAI2* gene is found to have higher significance in a temporal dataset of both classes and stands on top of the other genes, as depicted in Fig. 7. This gene influence the predictions of AD and non-AD temporal samples with a higher score alongside the subordinate genes.

## Discussion

Transcriptomic studies unravel novel insights into a clinical condition. This study is designed in a three-fold pattern (Fig. 1). The gene expression data is split into subgroups by the blood samples collected from three brain regions. Frontal, hippocampal, and temporal classes are grouped with control samples extracted from the same region. The frontal dataset contains 15 AD and 18 non-AD samples; the hippocampal dataset has eight AD and ten non-AD samples, and the temporal dataset with 10 AD and 19 non-AD samples. The most differentially expressed genes are extracted in the first fold. This step is crucial to finding informative genes from large-dimensional feature sets. The second fold identifies the candidate genetic markers from the DEGs with a genetic algorithm. 34 frontal, 60 hippocampal, and 28 temporal genes are strongly associated with AD. *ORAI2* (ORAI calcium release-activated calcium modulator 2) is the standard marker in all the candidate subsets (Table 1 and Fig. 2). Recent studies confirm the gene susceptibility of *ORAI2* with AD progression (Scremin et al. 2020; Ma et al. 2021). *TPI1* is another gene in both hippocampal and temporal markers (Fig. 2 and Table 1). The *TPI1* gene encodes instructions for the production of an enzyme known as triosephosphate isomerase 1. This enzyme is involved in glycolysis, a critical energy-producing process. *TPI1* was discovered through proteomics analysis to be a novel biomarker for predicting intrahepatic cholangiocarcinoma recurrence (Yu et al. 2020).

**Fig. 8:**
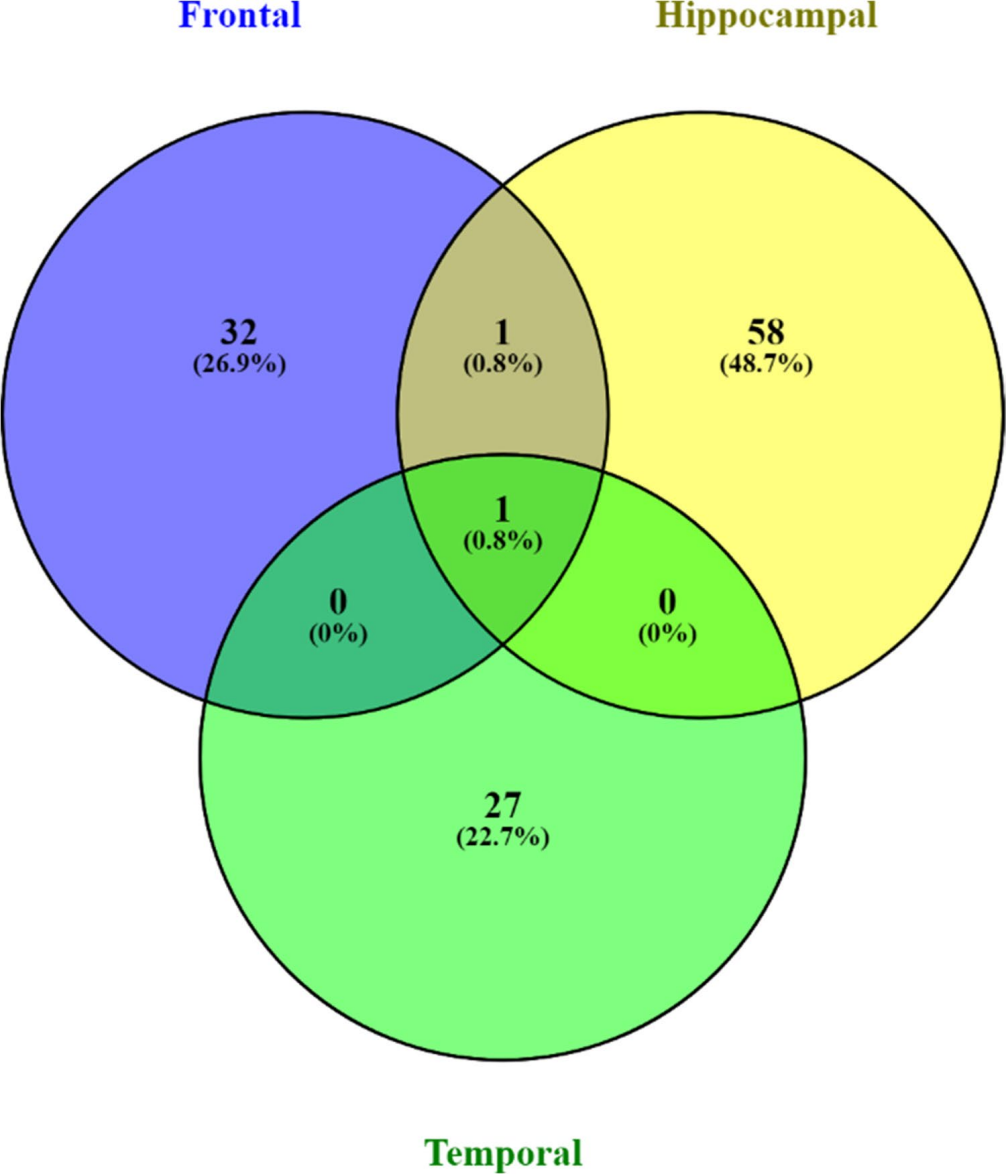
Overlapping gene ratio plot using Venn diagram

Figure 8 visualizes the overlapping genes identified from the candidate gene subsets. The *ORAI2* gene is present in all three datasets, and *TPI1* is found in frontal and hippocampal datasets. The *ORAI2* and *TPI1* genes are further analyzed to trace the genetic coalition linked to other AD-related disease markers (Fig. 2). Cluster analysis is performed on similar group genes by the k-means algorithm with 3 clusters. *STIM1*, *TRCP1*, and *ITPR1* genes directly impact neurodegenerative conditions (Fig. 2). TRP channels are the potential therapeutic targets for Alzheimer's and related illnesses (Yamamoto et al. 2007; Datta et al. 2020; Hwang et al. 2021). Also, mutations in these genes are found to have pathological relevance to AD. *TRPC6*, a dominant pathogenic gene, is known for the early onset of AD, which is strongly influenced by the mutation caused by *APP* or *PS1* genes (Dahlgren et al. 2002; Dillen and Annaert 2006). The molecular interactions between the *ORAI2* and *TPI1* are represented as a gene network using the genemania web tool, a Cytoscape plug-in available for association studies (Fig. 2). The biomarkers are mapped to the correlated genes most directly or partially associated with AD. *TRPC6*, *ORAI2*, and *STIM2* are evident, playing a significant role in AD pathogenesis and the regulation of Store-operated calcium entry (SOCE). These genes also have pharmacological properties in drug discovery activities (Hunanyan et al. 2021).

The third fold of this experiment evaluates the prominence of the identified candidate markers using machine-learning models (Fig. 3). Five classification algorithms are trained with the reduced dataset: naive Bayes, linear support vector machines, multilayered perceptron backpropagation, logistic regression, and random forest. The five-fold cross-validation method is chosen to train and validate the model performance (Fig. 3). The model scores are calculated with precision, recall, F1-score, accuracy, and MCC. The DEGs and GA-identified subsets are separately trained. As expected, the scores of DEGs are lower compared to GA markers. The irrelevant features from the DEGs are eliminated during the GA selection process.

The performance of ML models differs in every dataset, with different scores from each model. The naive Bayes algorithm performs better on all the datasets, scoring 90.9%, 96.9%, 94.4%, 100%, 93.1%, and 100% on frontal-DEG's-GA, hippocampal-DEGs-GA, and temporal-DEGs-GA respectively (Table 2). The higher scores of the models strengthen the genetic evidence found as a prominent marker of AD. The increase in performance displays the model's ability to discriminate the case and control AD samples. The dataset is minimally imbalanced over the binary target classes. The most reliable statistical metric, MCC, is calculated to avoid prediction bias by considering all four components of the confusion matrix—true positive, false positive, true negative, and false negative. Besides, precision, recall, and f1-scores ensure the robustness of the model evaluation. Surprisingly, the GA-identified subset performance against all ML classifiers in the hippocampal dataset is 100% in all calculated metrics. This evident finding exhibits the complete association of the genetic markers with AD. In temporal-GA, the NB classifier attained 100% performance in all scoring criteria, outperforming other classification algorithms. Apart from the random forest model, all other classifiers displayed the same performance on the frontal-GA dataset. In an evident study, nine lncRNAs named LncSigAD9 discriminate AD and healthy samples with higher sensitivity and specificity at 86.3% and 89.5%, respectively, with receiver operating characteristic curves of 0.863 (Zhou et al. 2019). A similar study with lncRNA and microRNA analyzed the expression patterns and revealed essential dysregulation genes. The investigations showed 85% to 95% accuracy in biomarker detection with ML models (Garcia-Fonseca et al. 2021).

The interpretation of ML model output discloses the essential genes contributing to the accurate prediction of AD or Non-AD samples. The black-box processing of ML algorithms minimizes visibility and increases uncertainty. Explainable artificial intelligence techniques are built to uncover the ML model's internal processes. This experimental study implements the LIME and SHAP methods to discover the prediction pattern from the ML algorithms trained with the candidate genes. The single observation from both AD and non-AD for all three datasets is fetched randomly for evaluation. The logistic regression model is used to train the datasets with 75% samples from which the explanation for the predictions is extracted. The LR learner is selected because it performs better with binary classification problems. XAI interpretation analysis allows comprehension of the internal process of AI models and makes it easier to interpret the decision-making of AI systems. The algorithm can be retraced, and a high level of visibility minimizes the "black box" effect. The salient feature of XAI is enabling humans to trust the predictions, maintain a high level of performance, and have context-aware decision-making (Pawar et al. 2020). There exist many types of XAI models for disparate objectives. The XAI methods can be either model-specific or model-agnostic, intrinsic or post hoc, and local or global. In this study, the evaluation of the discriminative capacity of ML models is implemented by LIME and SHAP. These two XAI methods are model-agnostic and post hoc, applied after the model has been trained.

LIME identified *ORAI2*, *RAB6A*, and probe7981324 as important biomarkers to predict a sample into the frontal AD class. The feature value of *ORAI2* is 10.63, which shows a higher priority than other genes. probe7981324, probe7894213, *COX4I1* predicts frontal-non-AD. probe8015796, probe8063347, and *SYT5* are the predictor markers of hippocampal-AD and *IL1RL2*, probe8015796, and *PPP1R11* for hippocampal non-AD. *ORAI2* again stands on top in predicting temporal AD samples alongside *AP1S1* and *SYT13* genes. *ORAI2*, *APOBEC3F*, and probe7892609 genes contribute more to predicting temporal non-AD cases. The SHAP model displayed its depth interpretation of the LR model prediction on different samples. The base value in a SHAP output represents the average predictions made by the model on the training dataset. The output value is the model-predicted value for the sample. For a random sample of temporal AD class, SHAP identified *RAB33A* influencing the prediction to 1, but *ORAI2*, *RAB6A* to 0. This interpretation proves the prediction as accurate that temporal AD is mapped to class 0 and temporal non-AD as 1.

*RAB6A* positively impacts predicting the sample as 0, but probe7981324, and probe7894213 to class 1, ensuring the genes contributing to the predictions and their closeness. 8,015,796 is more promising for hippocampal AD, and for hippocampal non-AD, *IL1RL2* is found as the biomarker. *ORAI2* is a common gene involved in predicting both temporal AD and non-AD. The identified biomarkers could act as targeted therapeutics for AD. The XAI models unveil profound insights by mining the prediction patterns of machine learning models. Further studies on the association between genes and disease through XAI techniques deliver promising findings and improve treatment outcomes.

## Supplementary Information

Below is the link to the electronic supplementary material.

Supplementary file1 Frontal-Biomarkers-GA information (CSV 10 KB)

Supplementary file2 Hippocampal-Biomarkers-GA information (CSV 9 KB)

Supplementary file3 Temporal-Biomarkers-GA information (CSV 7 KB)

## Data Availability

The data are available with the corresponding author GPDC.

## Code availability

The source code of the implementation is deposited in the GitHub repository. Link: https://github.com/karthiksekaran/alzheimer-biomarker-study-XAI
